# Probabilistic models of biological enzymatic polymerization

**DOI:** 10.1371/journal.pone.0244858

**Published:** 2021-01-06

**Authors:** Marshall Hampton, Miranda Galey, Clara Smoniewski, Sara L. Zimmer

**Affiliations:** 1 Department of Mathematics and Statistics, University of Minnesota Duluth, Duluth, MN, United States of America; 2 Integrated Biosciences Program, University of Minnesota, Duluth, MN, United States of America; 3 Department of Biomedical Sciences, University of Minnesota Medical School Duluth Campus, Duluth, MN, United States of America; Nippon Medical School, JAPAN

## Abstract

In this study, hierarchies of probabilistic models are evaluated for their ability to characterize the untemplated addition of adenine and uracil to the 3’ ends of mitochondrial mRNAs of the human pathogen *Trypanosoma brucei*, and for their generative abilities to reproduce populations of these untemplated adenine/uridine “tails”. We determined the most ideal Hidden Markov Models (HMMs) for this biological system. While our HMMs were not able to generatively reproduce the length distribution of the tails, they fared better in reproducing nucleotide composition aspects of the tail populations. The HMMs robustly identified distinct states of nucleotide addition that correlate to experimentally verified tail nucleotide composition differences. However they also identified a surprising subclass of tails among the ND1 gene transcript populations that is unexpected given the current idea of sequential enzymatic action of untemplated tail addition in this system. Therefore, these models can not only be utilized to reflect biological states that we already know about, they can also identify hypotheses to be experimentally tested. Finally, our HMMs supplied a way to correct a portion of the sequencing errors present in our data. Importantly, these models constitute rare simple pedagogical examples of applied bioinformatic HMMs, due to their binary emissions.

## 1 Introduction

In this paper, the framework of Hidden Markov Models (HMMs) was applied to an interesting data set from molecular biology. Our analysis had two major purposes. We wanted to identify strengths and weaknesses of HMMs in discovery and predictive roles for this specific dataset, and highlight the pedagogical utility of this dataset in teaching and exploring HMMs. A HMM is a probabilistic model consisting of a set of ‘hidden’ states which stochastically transition between each other with fixed transition probabilities; each state also stochastically emits observables with fixed emission probabilities. The hidden states are generated through a Markov chain process, with observables chosen randomly according to a state-specific distribution of probabilities. Historically one of the first uses of HMMs was in the field of speech recognition, and more generally language processing. Indeed Markov himself first used the simpler Markov chain formalism to explore patterns of vowels and consonants in Pushkin’s grand poem *Eugene Onegin*. These applications, while very interesting, are also very complex and are essentially impossible to analyze by hand. With the advent of computers, efficient algorithms for training a HMM on data (the Baum-Welch algorithm) and determining the most probable sequence of hidden states through a HMM given an emission symbol sequence (the Viterbi algorithm) were developed in the 1960s.

In the 1980s and 1990s the use of HMMs exploded in popularity in bioinformatics applications, mainly for analyzing DNA or protein sequence data. A classic example is the GENSCAN gene-finding algorithm of Burge and Karlin [1], which is also quite complex. The introductory examples of HMMs in bioinformatics textbooks are usually quite artificial because of the complexity of emission variables in most natural examples in molecular biology. The problem we consider here has only two emission observables, and so provides a novel and non-contrived setting for simple HMMs with real biological content. The famous text of Durbin, Eddy, Krogh, and Mitchison [2] uses the occurrence of CG islands to introduce HMMs, but even this relatively simple model requires a minimum of eight hidden states and four emission types. In contrast, the most complicated models we show here have six states and only nucleotides adenine (A) and uridine (U) as emission types.

The mRNA of almost all eukaryotes is modified from its original transcribed sequence by non-templated nucleotide addition (typically polyadenylation, or addition of successive adenosines (As)) to the 3’ end. This occurs in both cell nuclei and in organelles that possess independent genomes. The length of a 3’ poly-A tail varies greatly among species and transcripts. In humans most non-mitochondrial transcripts have tails of 40-80 As, although the full range is 0 (for some histones, for example, which are polyuridylated [3]) to at least 250 bases [4]. Our focus is the unusual 3’ addition of both A and U to mitochondrial transcripts of the human parasite *Trypanosoma brucei*. Some addition of U to poly-A mRNA tails has been described in other systems, for example in myxomycetes (*Stemonitis flavogenita* and *Physarum polycephalum*) [5], yeast (*Saccharomyces pombe*) [6], plants, and algae and plants organelles [7–10], and humans [3]. In *S. pombe* the uracil additions can affect degradation pathways [11]. However the complexity and role of these additions seem limited compared to those in the mitochondria of *T. brucei* and other Kinetoplastida. Of all natural non-templated nucleotide addition processes, those in the kinetoplasts may be the best suited to explore dual emission type HMM.

The order Kinetoplastida (NCBI taxonomic id 5653) consists of hundreds of species, some of which are heteroxenous parasites. Some insect-transmitted trypanosomes, including *Trypanosoma brucei*, which causes African sleeping sickness in humans and nagana in cattle, threaten the health of humans and livestock. The kinetoplastids are characterized by an unusual single mitochondrion containing an extraordinarily large amount of DNA [12] the expression of which requires multiple novel post-transcriptional events [13]. In addition to the previously-mentioned addition of non-templated tails consisting of A and U, most of the mitochondrial mRNAs undergo a targeted insertion and deletion of a few to hundreds of uracils (RNA editing) to generate a translatable sequence [14]. For transcripts undergoing editing, their identity as pre-edited or edited will influence the nucleotide composition of their tail populations. An initial extension (an in-tail) with particular characteristics including a high composition of A is initially added to each gene’s transcript population. However, transcripts that do not require editing to encode their protein, or those in which editing has been completed, can acquire an extension to the in-tail and become an ex-tail with a higher composition of U. Ex-tails are not present on transcripts prior to editing [13]. An example is shown below.

$$\begin{array}{c} \underset{\text{templated}}{\underset{︸}{\ldots \text{GCUAGG}}}\underset{\text{untemplated}\;\text{tail}}{\underset{︸}{\overset{\text{in-tail}}{\overset{︷}{\text{UUUAAAAAAAAAAAAAAAAAA}}}\overset{\text{ex-tail}}{\overset{︷}{\text{UAAUUAAUAAAAUUAAUAUAU}}}}} \end{array}$$

In *T. brucei* the respiratory pathway that is in part encoded in the mitochondrial genome is essential for its survival in the insect but shut off when *T. brucei* is in the glucose-rich bloodstream [15–17]. Remodeling of mitochondrial gene expression occurs as part of this transition [18]. At least some regulation of the mitochondrial transcriptome occurs at the RNA level [13], and we have previously analyzed the variation of content (nucleotide length and composition) in the 3’ tail additions between life stages. We have found differences in tail composition between insect (P = procyclic) and bloodstream (B= (mammalian) bloodstream) life stages [18]. Other studiess have identified relationships between tail presence and stability [19, 20], tail composition and precursor mRNA processing [20], and tails and translation [21, 22].

Uridylation of Kinetoplastida mitochondrial transcripts is primarily performed by the protein KRET1 [23], and adenylation by KPAP1 [19], that are both members of protein complexes. A host of RNA binding proteins of the PPR family modulate tail addition and stability [20, 22, 24–26], and putative enzymes such as KPAP2 [27] may also play roles. Deciphering the mechanism of and roles for 3’ tail additions in *T. brucei* has required genetic manipulation and subsequent tracking of downstream effects such as mRNA tail composition.

This approach has proven hugely informative, but mechanisms of tail addition are clearly complex. We undertook the current study in part to determine what is additionally gained by analyzing 3’ tail addition from the opposite orientation. We wished to know if features inherent in tail populations could reveal additional information about the process of untemplated addition that did not become apparent when we used a biologically-guided choice of model [18]. We also wanted to assess the capacity of HMMs for predicting non-templated tail features, including computationally parsing and potentially correcting any biases inherent in the use or manipulation of Illumina sequencing data to characterize tail populations. The results of our study apply to all non-encoded nucleotide synthesis that is not restricted to single-nucleotide homopolymer addition. They also highlight a simple context for HMM that is of potential pedagogical benefit.

## 2 Statistical properties of tail populations

Our current and previous [18, 28] work focuses on a dataset of tail populations from transcripts of the *T. brucei* mitochondrial genes CO1, CO3, and ND1 collected from both the procyclic and bloodstream life stages (denoted CO1B, CO1P, CO3B, CO3P, ND1B, and ND1P henceforth). Unlike CO1 and ND1, CO3 is an edited mRNA. The CO3 tails analyzed here are derived from the pre-edited CO3 transcripts only, and thus are entirely in-tailed. CO1B transcripts also lack ex-tails because even while these mRNAs exist in low abundances in bloodstream-form cells, they are very likely not translated and indeed lack evidence of ex-tails [18]. Statistical properties of these tail sequences were presented in previous publications [18, 28], including the distribution of tail lengths and adenine content. However, before aggressively evaluating the full potential for HMMs in defining and comparing these datasets, we wished to perform two additional statistical analyses, as this information could be useful in interpreting any unusual or unexpected models that we might later encounter.

**Fig 1:**
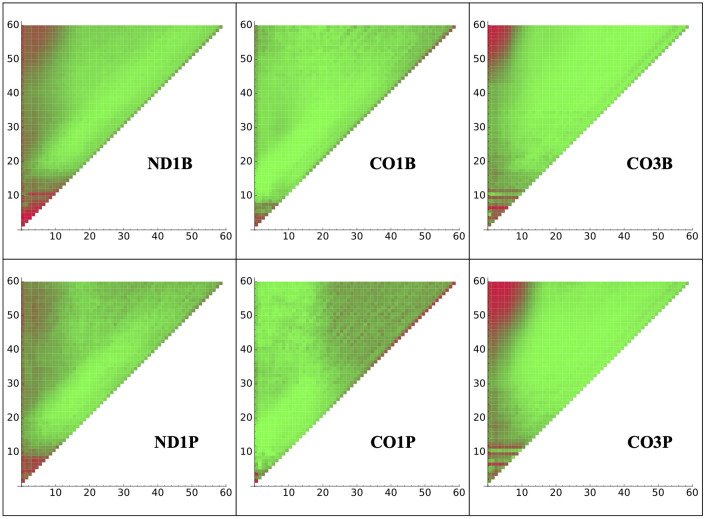
Tail composition by length. U/A composition of tails at each nucleotide position indicated on the x axis (Position 1 is the first non-encoded nucleotide attached to the mRNA’s 3’ end) for the population of tails of exactly each length specified on the y axis. Red and green indicate 100 percent U and 100 percent A, respectively.

It was important to determine if an early stage of tail addition influences subsequent nucleotide addition. One pattern that would suggest such a process would be if predominant nucleotide identity (A or U) of positions early in the tail differed for tails of specific lengths. An indicator of such an influence would be differences in composition of the early tail sequence between tails of different lengths. Therefore, we analyzed the positional composition of tail populations of fixed lengths from 1 to 60 nucleotides. For CO3 tails, a strong correlation between beginning with a U-rich region and tails that achieved lengths of ≈ 50 nucleotides or more was observed. A heat map of A versus U composition for each position specific to populations of discrete tail lengths is shown in Fig 1 for all analyzed tail sets. This result suggests that the composition of nucleotides added early in tail addition can affect the number of subsequent additions.

**Fig 2:**
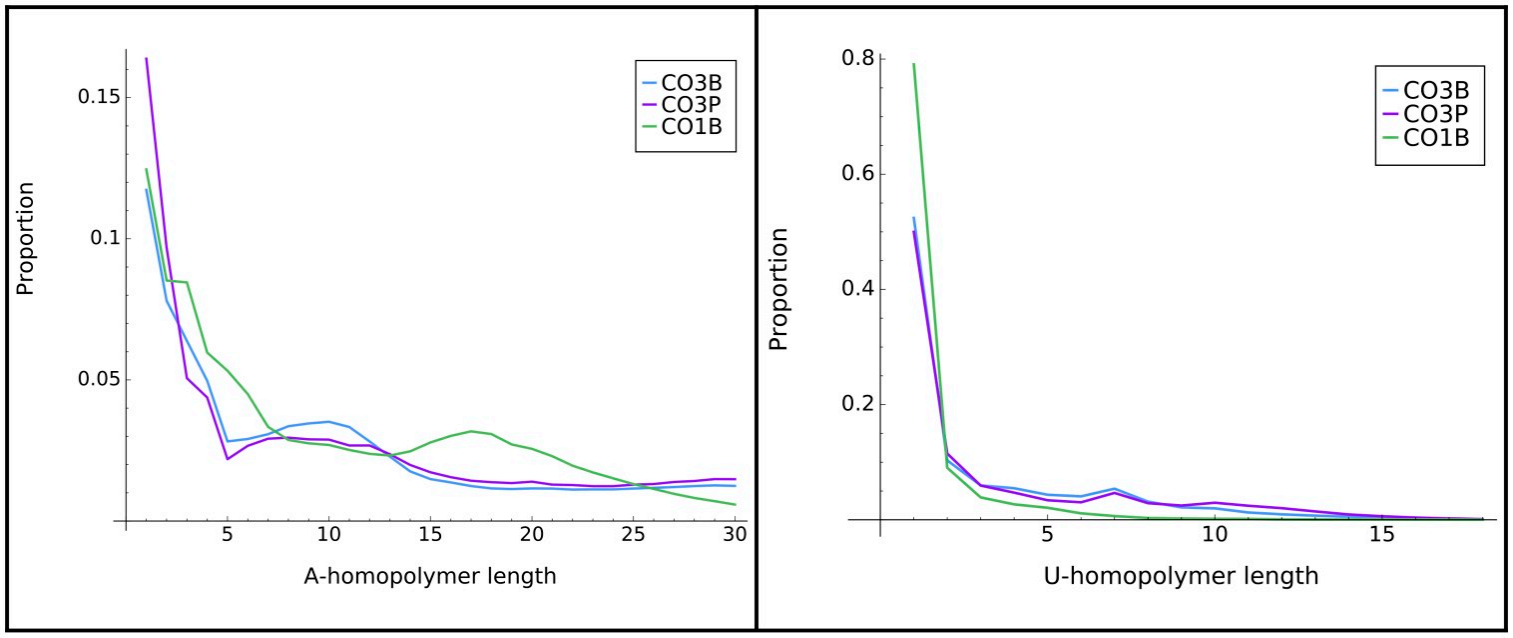
Homopolymer distribution in tails. Distribution of lengths of A-homopolymer (left) and U-homopolymer (right) found within tail populations of the three transcripts that are only expected to possess the primarily homopolymer-containing in-tails. Y axis scales differ in the left and right graphs.

We also analyzed a feature that is a hallmark of in-tails: a preponderance of homopolymeric additions (either A or U), in comparison to the more frequent nucleotide switching observed in ex-tails (see example in Introduction). To specifically examine in-tails we utilized the tail population datasets that lack ex-tails for this investigation (those derived from CO1B, CO3B, and CO3P) (Fig 2). We first examined differences in A and U polymer lengths between transcripts. We found that the distributions of U-homopolymers were simpler than that of the A-homopolymers, with almost entirely monotonic decreases. Almost all U-homopolymers were less than 15 bases long for all examined tail populations. The A-homopolymer distribution was more complicated in that the distribution contains more robust, well-defined peaks of longer homopolymers. The CO3B and CO3P tail population distributions are more similar to each other than those of CO3B and CO1B. This provides additional indirect evidence that in-tail additions are regulated differentially between genes, as suggested in earlier works [18, 28, 29].

The previous result led us to focus predominantly on the distribution patterns of A-homopolymer rather than U-homopolymer additions within each population of tails. Initially we selected the CO1 populations for which to perform this analysis, because CO1 shows both ex-tail addition (in the procyclic form) and virtually no ex-tails in the bloodstream form. In enumerating homopolymers, we developed an index that refers to homopolymers of both A and U numbered from 5’ to 3’, as shown:

$$\begin{array}{c} \overset{\text{A1}}{\overset{︷}{\text{AAAA}}}\underset{\text{U2}}{\underset{︸}{\text{UUUUUU}}}\overset{\text{A3}}{\overset{︷}{\text{AAA}}}\underset{\text{U4}}{\underset{︸}{\text{UUUUUUU}}}\overset{\text{A5}}{\overset{︷}{\text{AAAAAAAAAAAAAAAA}}}\ldots \end{array}$$

and

$$\begin{array}{c} \overset{\text{U1}}{\overset{︷}{\text{UUU}}}\underset{\text{A2}}{\underset{︸}{\text{AAAAAAAAAA}}}\overset{\text{U3}}{\overset{︷}{\text{UU}}}\underset{\text{A4}}{\underset{︸}{\text{AAAAAA}}}\overset{\text{U5}}{\overset{︷}{\text{UUUU}}}\ldots \end{array}$$

Originally, tail data was acquired by individually cloning and sequencing tails in limited numbers, so that only the most predominant tails were captured. From that time, in-tails have typically been described in the literature as A-homopolymers, sometimes possessing an initial addition of a short U-homopolymer, as these tails predominate. Therefore, one point of concern was that quantitation of in-tail homopolymers at the level of A/U3 and higher would be very rare relative to the A-homopolymer (A1) tails of the population, and thus their length distributions supported by very few reads. However, as the total number of tails analyzed was so high, this was not the case. For example, over 58,000 tails were used to compute the A5 value for CO1P tail population.

**Fig 3:**
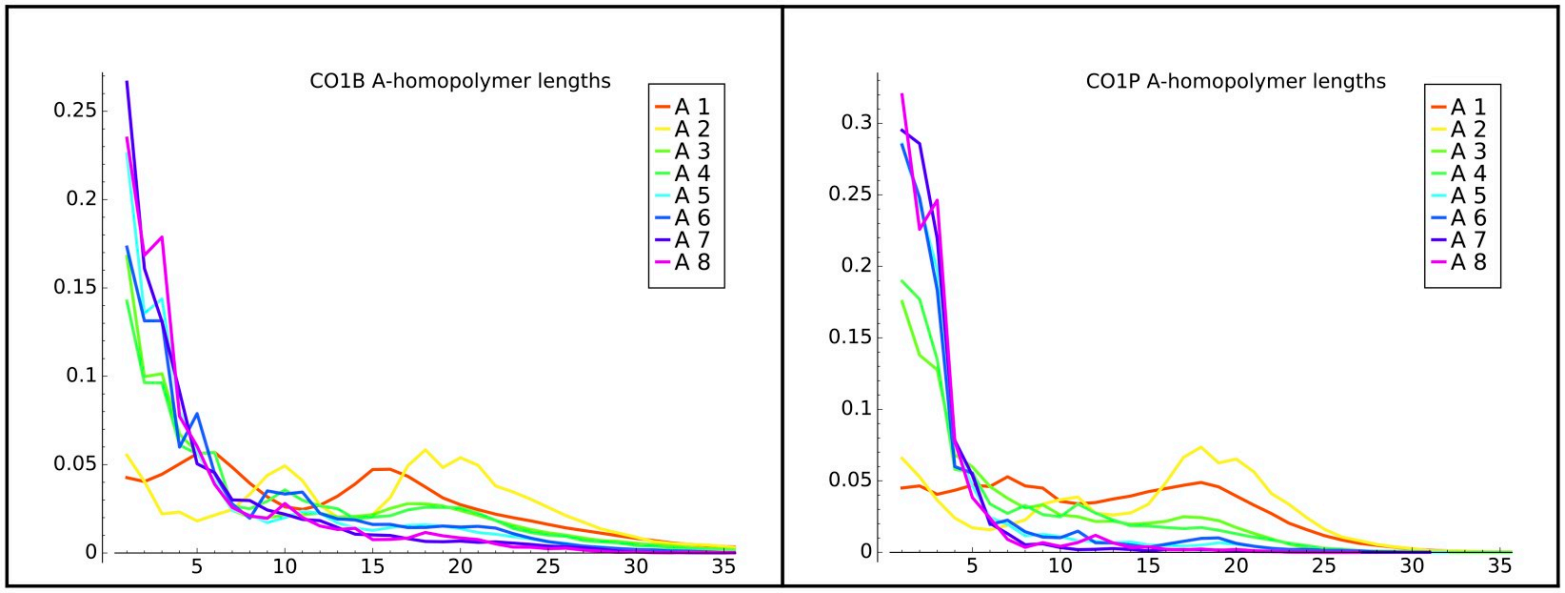
CO1B and CO1P A-homopolymer distributions. The frequency distribution of lengths of A-homopolymers in CO1B and CO1P transcript tail populations. A1 indicates the first homopolymer encountered starting from the first nucleotide of non-templated addition, A2 indicates the second, on to the eighth encountered homopolymer (A8), in the populations of tails in which they occur.

**Fig 4:**
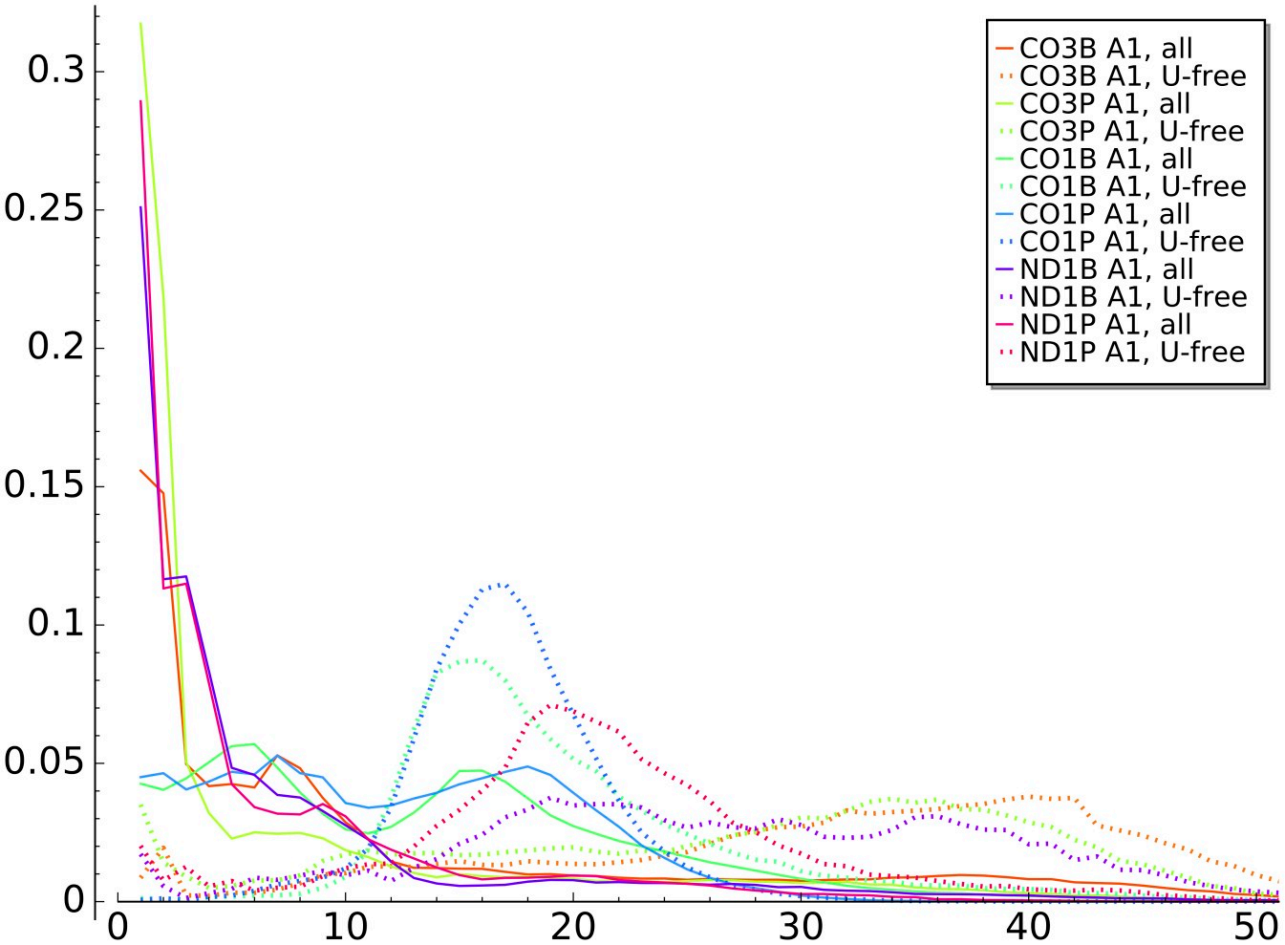
Initial A-homopolymer distributions. Distribution of initial A-homopolymer lengths for tail populations of all analyzed transcripts (solid lines) and poly(A) tail-only sub-populations (dotted lines) for each transcript tail dataset.

We found that the length of the first A-homopolymer can vary from later homopolymers. Specifically, the initial A-homopolymer (A1 or A2) is considerably longer in the CO1 gene in both life stages, as shown by the peaks around 20-25 bases in Fig 3. This result might reflect that initial A-homopolymer addition is in a different biological context than later in-tail additions. Interestingly, the distributions of the initial (A1) homopolymer in other genes (CO3P and ND1) in either life stage do not exhibit a trend similar to the CO1 A1 homopolymer. These transcript tail A1 datasets do not show spikes of longer homopolymers in Fig 4 (solid lines).

We hypothesized that the cluster of CO1 A1 homopolymers in the range of 15-30 nt in length may be largely comprised of tails containing no U (exclusively poly(A)) that are traditionally considered the initially added tail in trypanosome mitochondria. To determine this, we analyzed only sequences with no Us in them (i.e. there is no U1 state; a poly(A) tail) and compared the distribution of A-homopolymer lengths to those of all A1 homopolymers. We did this for all transcript sample sets to determine whether this is true only of CO1 tail populations or is a larger trend most visible among the CO1 tails. The length distributions are strikingly different, as the poly(A)-only tails appear much more precisely length-controlled than is A addition in the context of co-occurring oligouridylation (Fig 4). While this is most obvious for CO1 populations, it is also evident to a lesser extent for the ND1P tail population. Because of the difference in homopolymer lengths between the A1 and later A-additions, and the higher length control exercised on a poly(A) tail, in our final models (described in Section 5, below) we added a separate progressive state (‘A-only state’) that only adds the initial A1, which we will describe at that time. Also uncovered by this methodology are a longer subpopulation of poly(A) tails in the 25-45 nt range on other gene transcript populations including those of the pre-edited CO3 tails.

To provide a sense of the differences between the tail sequences that we seek to capture in our models, we show below three randomly selected sequences from each tail population, drawn from the population of sequences that only appear on mRNAs of that specific gene and life-stage.

CO3B:

UUUAAAAAAAAUUUAAAAAAAAAAAAAAAAAAUAAAAAAAAAAAAAAAAAAAAAAAAAA

UUUUUUUAAAAAAAAAAAAAAAAAAAAAAAAAAAAAAAUAAAAAAAAAAAAAAAAAAAA

UUUUUUUAAAAAAAAAUAAAAAAAAAAAAAAAAAAAAAAAAAAAAAAAAAAAAUAAAAAAUAA

CO3P:

UUUUUUUUUUUUUUUUUAAAAAAUAAAAUAAAAAAAAAAA

AUUUUUUUUUUUUUUAAAAAAAAAAAAUAAAAAAAAAAAAAAAAAAUAAAA

AUUUUUUUUUUAAAAUAAAAAAUAAAAAAAAAAAAAAAAAAAAAAAA

CO1B:

AAAAAAAAUAAAAUAAAAAAAAAAAAAAAAAAAAAU

AAAAAAAAAAAAAAAAAAAAAAAAAAAAAAAAAAAUAAAAAAAAAAUUUUUAUU

AAAAAAUAAAAAAAAAAUAAAAAAAAAAAAAUA

CO1P:

AAAAAAAAAAAAAAUUAAUAAAAAAUUAAAAAUUAAAAUAUAAUAAUAAAAUAU

AAAAAAAAAAAAUAAAAAAAAUAAUAAAUAAUAUAUAAUAAUUAAUUUAAUUUUUUAUAUAU

AAAAAAAAAAAAAAAAAAAAAAAUAAUUAAUAAAAUUAAUAUAUAUAAUAAUA

ND1B:

AAAUAUUUUUUAAAAAAAAAAAAAAAAAAAAAAAAUUAAA

UUUUUUUUUAAAAUAAAAAAAAAUUUUUUUAAAAAAAAUAAAAAUUAAAAAAAAAAAAAAAAAAAA

UUUUUUUUUUUUUUUUUUUUAAAAAAAAAUUUUUAUUAAAAAAAAAAUAAAAAAUAAAAAAAU

ND1P:

AAAUUUAAAAAAAAAAAUAAAAAAAAAAUAAAA

AUAAUUAAAAAAAUAAAAAAAAAUAAAAUAAUAUAAAAUAAAAAUAUAAAAAAUAUAAAUUUAAAUAA

AUAAAAAAAAAAUAUUAAUAUUAAUUAUUUAUUAUAUAUAAUAUAAUAUAUAUUAAAAUUAU

## 3 Performance of biological system-informed HMMs of increasing complexity

**Fig 5:**
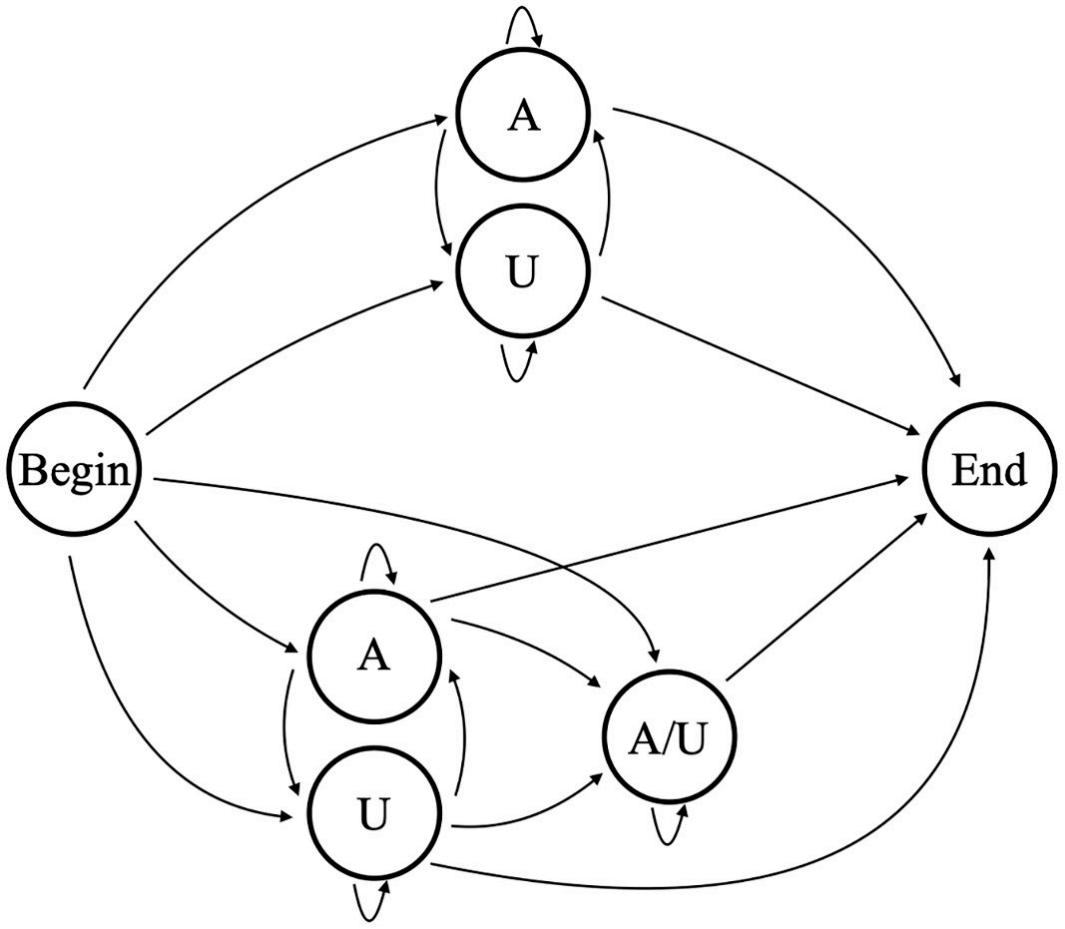
Model B5. 5-state model (B5) used to determine tail addition in previous studies.

In previous work [18] we categorized in-tails and ex-tails by using a HMM of one discrete complexity level. Our previous HMM [18] (in this text referred to as model B5, shown in Fig 5) contains five nucleotide-adding hidden states; a single one of those hidden states corresponds to the ex-tail addition. For a given sequence the Viterbi method was used to determine the most likely path through the model, and if the state path contained the ex-tail state the corresponding nucleotides were considered to be part of the ex-tail. Our 5-state HMM worked well for that categorization purpose, but in order to explore the generative ability of HMMs as applied to these datasets and potentially identify unexpected states, we considered HMMs of a range of complexities.

**Fig 6:**
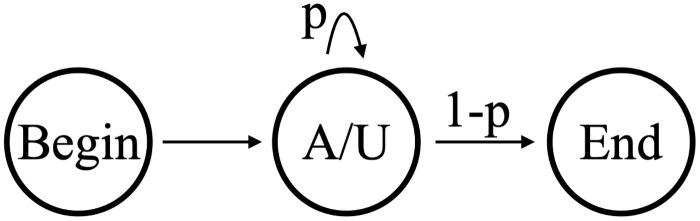
Model B1. The 1-state model (B1) of nontemplated nucleotide addition on *Trypanosoma brucei* mitochondrial mRNAs.

**Fig 7:**
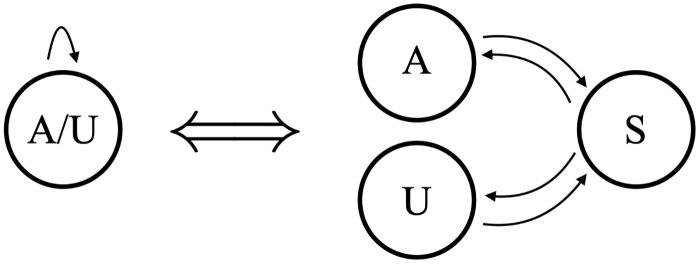
Model equivalence. Equivalence of mixed hidden states with a distributive process of nontemplated nucleotide addition.

The simplest possible model with which to analyze our datasets is shown in Fig 6. A single state emits either an A or a U, with the emission probabilities determined by the training data (the silent Begin/End states are ignored in our state counts). We will refer to this as Model B1 (Beginning model 1). Model B1 has 2 independent parameters: one determines the transition probabilities (p and 1-p in our figure) out of the emittive state, and the other determines the proportion of A versus U in the emissions. The hidden states that emit both A and U can be interpreted biologically as a distributive or a progressive process. As shown in Fig 7, a silent S state can represent a disassociated enzymatic complex rather than a continuous process of addition shown on the left. Computationally it is simpler, and equivalent, to model the processive addition of mixed emissive states. Regardless of this flexibility, Model B1’s output would be insufficient to capture the strong correlation between consecutively added nucleotides in the in-tails—i.e. the tendency to have longer homopolymers. Since the limited output of Model B1 is only theoretically sufficient to capture final ratios of A and U in the tails, we did not utilize it and examined the the next-simplest possibility instead.

**Fig 8:**
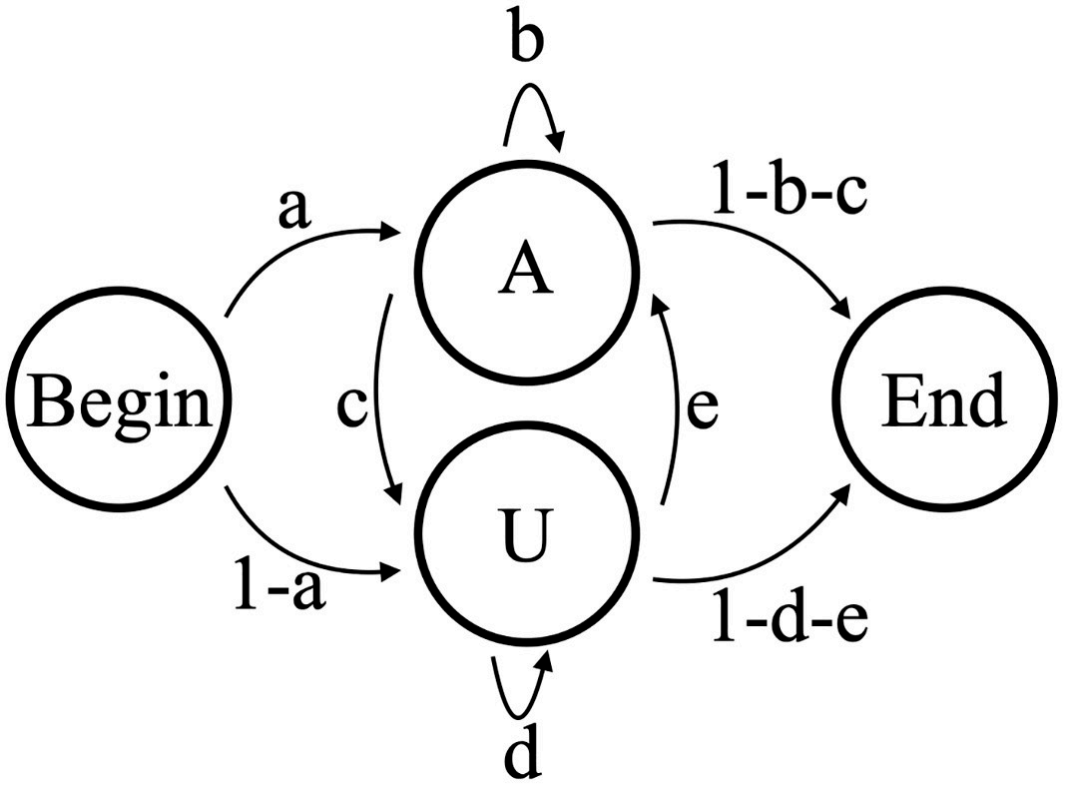
Model B2. 2-state model of nontemplated tail addition (B2), with five independent transition parameters a,b,c,d, and e. This model cannot be used to distinguish between in-tail and ex-tail addition.

A two-state model, B2, allows separate states for adding an A or U. Model B2 as shown has 5 independent parameters (Fig 8). This model would do a much better job than B1 at capturing the overall correlation between consecutively added nucleotides within the tail dataset. However, if subsets of tails within the complete dataset possess different or changing correlations, Model B2 would be inadequate to capture this. Specific to our datasets, Model B2 cannot distinguish which states correspond to the in-tail and which states correspond to the ex-tail addition.

**Fig 9:**
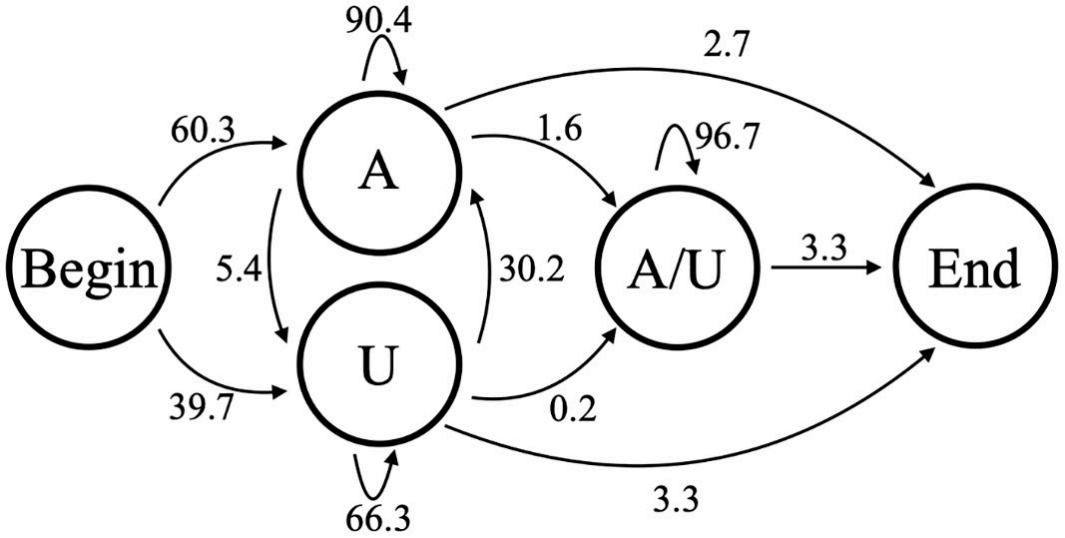
Model B3. 3-state model of nontemplated tail addition (B3), with state transition percentages from the model trained on combined CO1P, ND1B, and ND1P experimentally-derived Illumina sequenced tail data.

In contrast, Model B3 includes an additional state that would allow for modeling differences between in-tail and ex-tail characteristics if more than just the in-tail state exists for the tail population. It is the simplest possible model that can reflect dual states and has 9 independent parameters. Any tail passing through the state depicted as “A/U” in Fig 9 is considered an ex-tail. We generated a combined dataset of tail populations of the three transcripts whose tail populations should reflect ex-tailing on which to train this model. Fig 9 includes the emission probabilities after training on a combined ex-tail-containing dataset (samples CO1P, ND1B, and ND1P). In Model B3 and Model B5 described next, the ex-tail state adding both As and Us should train to have close to a 7:3 ratio, as observed experimentally [19] when such a state is biologically present. Indeed, in all samples which contain ex-tails, we found that the A/U state converges after training to a value approximating a 7:3 ratio (in some cases, the number was closer to a 2:1 ratio). In contrast, the A/U state from samples which do not contain ex-tails do not train to a similar A:U ratio. Instead, this potential A/U state usually converges an A-adding state. Tails which are modeled as passing through the ex-tail A/U state are approximately twice as long as those modeled as consisting entirely of in-tail sequence under both our B3 and B5 models. The assignment of a nucleotide to the ex-tail state is very robust for these models; in comparing the Viterbi algorithm assignments of states for models B3 and B5, over 99% of the tails passing through the B5 A/U state passed through the B3 A/U state.

One thing that could not be captured by Model B3 is whether there is information imparted in the in-tail that influences whether or not it will become ex-tailed. The correlation of an initial U sequence with longer tails in CO3 tail populations seen in Fig 1 is not relevant here because those populations contain only in-tails. To capture the possibility of ex-tail additions being specific to some characteristic of in-tail addition, a model would need a separate path for exclusively in-tail additions. Model B5, which we previously used to quantify tail features [18], possesses this feature. We performed an analysis for each tail population to see if the sequences going though the in-tail only top set of states were any different than those passing through the in-tail to ex-tail bottom set of states. We found no statistical differences between these, in terms of overall length or overall composition, for any of the six transcript datasets. In light of this, Model B3 is nearly as sufficient as Model B5 for capturing the characteristics of these tails, at least in the populations analyzed.

**Fig 10:**
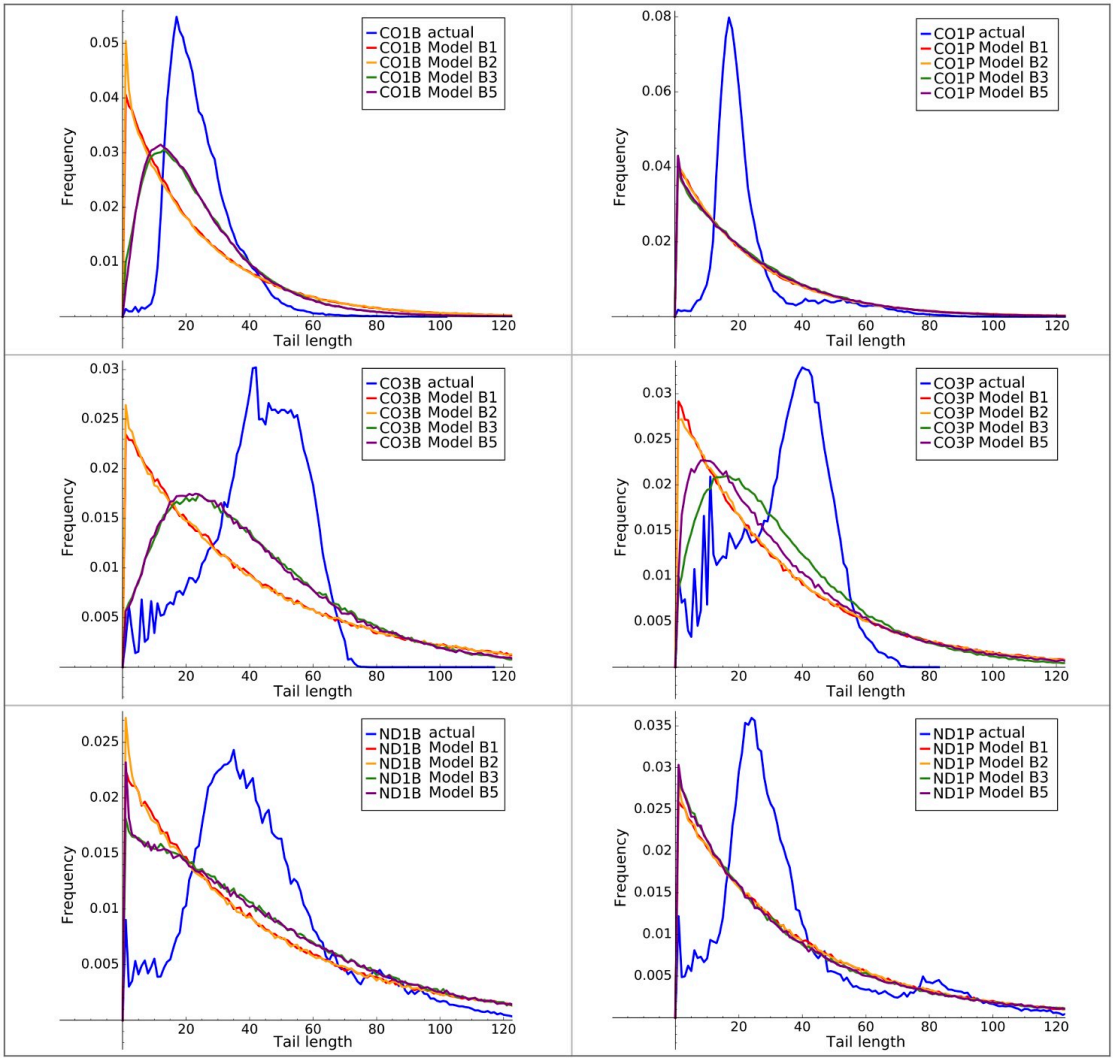
Actual and model tail length distributions. Observed and model output tail length distributions using B-series HMMs of all tested complexity levels.

The B3 and B5 models can classify in-tails and ex-tails, but we wanted to know their capacity as generative models. For each of our six tail datasets, we generated a tail length frequency profile using Models B1 through B5 trained on each respective dataset. The mean tail lengths generated from every model were very close to the observed mean length (in every case the difference in mean length was less than 5% of the standard deviation). However, these models failed in their ability to recapitulate the tail length distribution, as shown in Fig 10. In every case the standard deviation of tail lengths generated by the models is much larger than that of the observations.

As neither of the relatively simple HMMs B1 or B2 performed better than Model B3 or Model B5 at matching the details of the tail length distribution, we concluded that the termination of tail elongation is not a process that is well modeled by the memoryless Markov state structure. The empirical length distribution can be forced by *ad hoc* mechanisms but this fails to illuminate any additional features of biological relevance.

**Fig 11:**
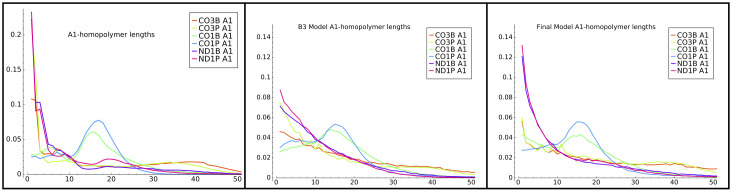
Model B3 A1-homopolymers. Distribution of initial (A1) homopolymer lengths generated by model B3.

We also examined the A and U homopolymer length distribution output by our models. For example, Fig 11 left and center panels compares the distribution of lengths of the initial (A1) A-homopolymers in our data and from model B3. The B3 model reproduced the differences in the CO1 lengths quite well—i.e. the local maxima at tails lengths of approximately 16 nucleotides, which are unique to that gene among the three studied. It also captured to some extent the presence of longer tails in the CO3 gene samples.

## 4 HMM sequence error correction

The success of the Model B3 in classifying the in-tail versus ex-tail states suggested that this model could also be used to correct for some sequencing and library-creation errors in our tail sequences. Prior to being used in training models, we had previously restricted the tail population datasets to tails only containing nucleotides A and U. Although this removed approximately 14 percent of the tails across all datasets, the datasets were so large (averaging over 700,000 tail sequences per sample type) that the tails they contained after clean-up still far exceeded the minimum number to adequately train our models and use the models to detect differences between tail populations. However, we wanted to see how our outputs would change if those eliminated tails were corrected and restored to the tail population.

CircTAIL-seq combines circular reverse transcription—polymerase chain reaction (cRT-PCR) and deep sequencing techniques, and the errors associated with each of these techniques need to be considered in the overall error rate. In our tail sequences, we have assumed that every C/G is an error. The reason for this is two-fold: (1) There have been no enzymes identified that can efficiently add C/G. While KPAP1 can inefficiently add C/Gs at high concentrations (100uM), KPAP1’s affinity for As exceeds this even at much lower concentrations (1uM) [19]. (2) While the As and Us have a non-random pattern in transcripts and life-stages, and between in- and ex- tails, the C/Gs have no discernible pattern. We acknowledge that we cannot determine if any of the A/Us are cRT-PCR or sequencing errors with this method. We also recognize that every C/G may not be a cRT-PCR or sequencing error, but we cannot differentiate between technique-derived errors and cell or enzyme-derived errors. KRET1 (the U-adding enzyme) has been shown to have a slight affinity for Cs rather than Gs [30], which led us to compare error rates of overall transcripts to transcripts with high numbers of Us. While there are slightly more C errors than G errors across all transcripts (C error rate = 0.00352, G error rate = 0.00262), when we examine only transcripts that have a high U content, the C and G error rates (C errors = 0.00358—0.00332, G errors = 0.00272—0.00247) do not change. This suggests that we cannot link the addition of C to KRET1 activity. If some instances of C/Gs are in fact the actual state of the sequences inside the cell, then the error rate for our process will be lower than what we here have calculated.

To decrease errors during library creation, the PCR step is optimized in circTAIL-seq by determining the fewest number of cycles possible while still generating a product [28]. Additionally, unlike other 3’ non-encoded tails, trypanosome tails are made up of heterogenous A/U sequences, which reduces, though does not negate, the concern for error associated with long homopolymeric regions in Illumina sequencing [4, 31]. The C/G error rate was 0.004 per base for tails with two or less C/Gs. We did not include the tails that had more than two C/Gs because they could represent tails that were not processed correctly and still include pieces of the transcripts from which they were derived. This C/G error rate includes 2/3 of the possible errors as each base has three incorrect options. We then multiplied the C/G error rate by 1.5 to determine an overall error rate of 0.006. Using the estimated error rates for the KAPA2G robust polymerase (5.88 ⋅ 10^−6^, KAPA Biosystems) and for MMLV polymerase (3.3 ⋅ 10^−5^ [32]), and the equation supplied in [33], we found the RT-PCR step had an estimated error rate of 0.000143. Next, we investigated the error rate associated with Illumina sequencing by analyzing the error rate for the PhiX DNA that was spiked in before sequencing. This error rate was 0.0048, so we determined that the majority of the errors were Illumina sequencing related. This source of error is unavoidable, so we considered methods to overcome these errors.

**Fig 12:**
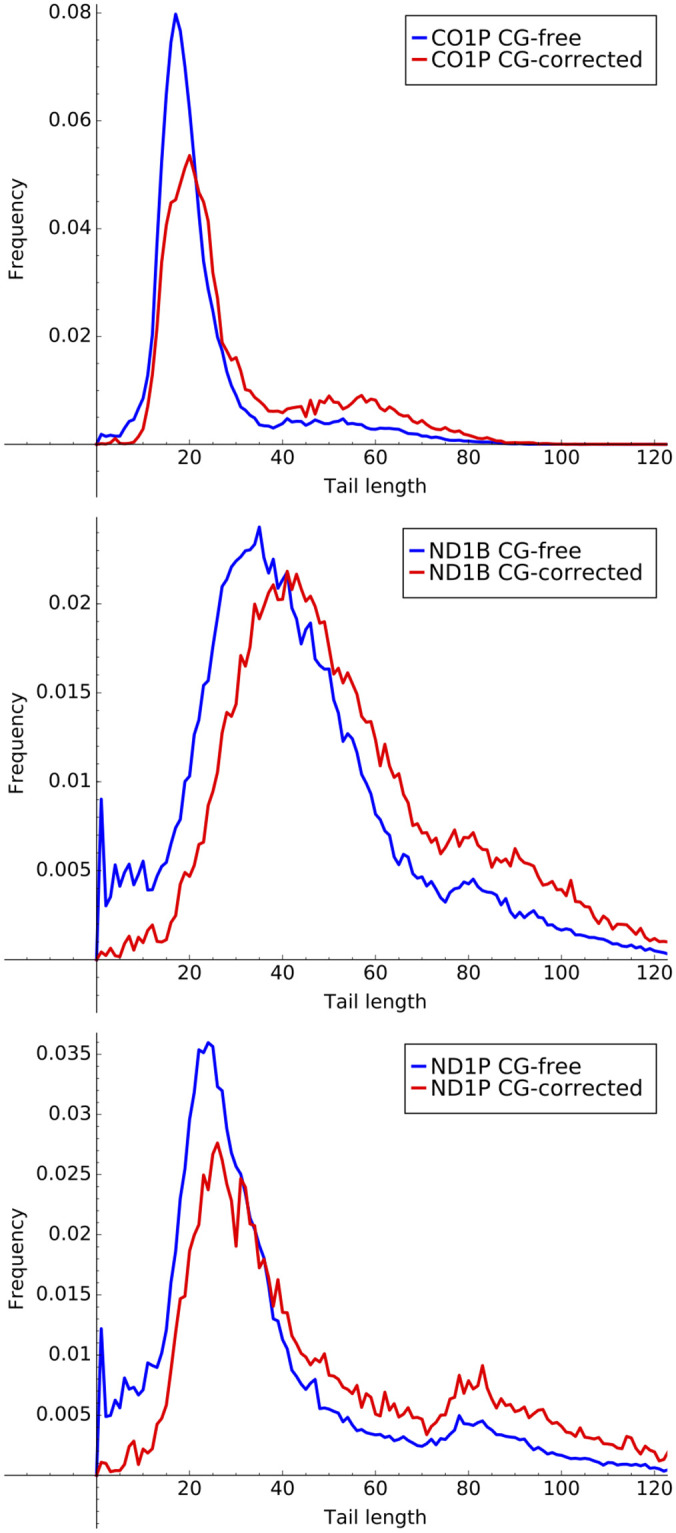
Error corrected tail lengths. Histograms of tail lengths for ex-tail containing datasets CO1P, ND1P, and ND1B inclusive and exclusive of sequences containing corrected G and C erroneous nucleotides.

12% of our sequences from analysis across all datasets contained a single C or G, corresponding to an error rate of 0.0035 per letter. We extended the emissions of Models B2 as well as Model B3 to include Cs and Gs, and then corrected the Cs and Gs based on the Viterbi algorithm’s state assignment for each tail sequence. For Model B3, the corrections for the ex-tail state with approximately 70% A and 30% U emissions were randomly re-labeled with the same probabilities as the emissions. Because errors were more common in longer sequences, the main result of our corrections was an upward shift in the observed tail length distribution as shown for the three genes with transcript tails populations containing ex-tails in Fig 12. Finally, we considered attempting to correct for errors in the A and U as well as the G and C bases. However, any such correction would be much more dependent on knowledge of the true biological tail addition process, and so we deferred this until more experimental data is available.

## 5 Unstructured and ultimate HMMs

**Fig 13:**
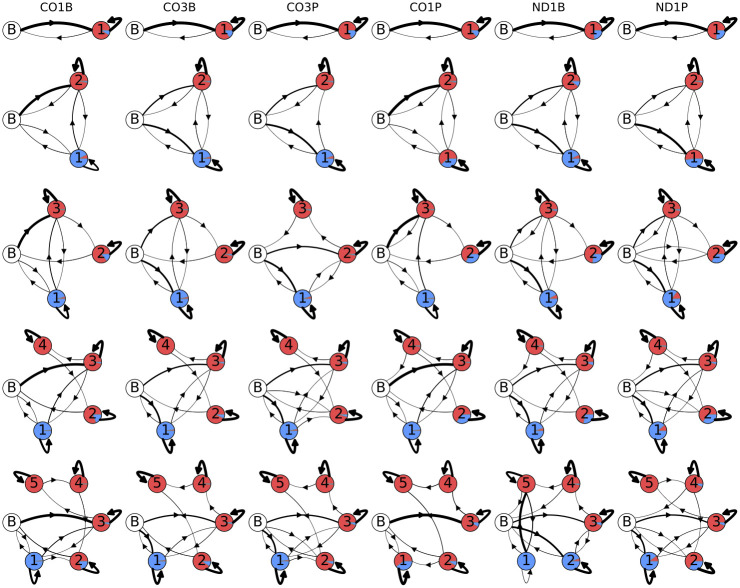
Unstructured model topologies. Post-training unstructured model topologies and emissions of U and A nontemplated tail addition to populations of 3’ mRNA ends of the mitochondrial genes indicated at the top. Models go from simplest in the top row (G1, 1 state) to complex at the bottom (G5, 5 states). The areas covered by the separate colors in each state circle are proportional to their emissions: uncolored circle labeled ‘B’ indicates the beginning/end state, red is single adenine addition, and blue is single uracil addition. The thickness of the arrows connecting states is proportional to the transition probability.

Although there is a logical, biological basis for the structure of the models we have been using thus far, by structuring them as we have we have potentially introduced some bias and missed discovering state possibilities. We therefore examined unstructured (designated with a G)) models which had no restrictions on their emissions and transitions, apart from having a single well-defined start/stop state. These models (G1—G5), with an expanded complexity range relative to Models B1—B5, are shown in Fig 13. Initial transition probabilities were chosen for each gene and life stage to match the observed A1 distribution. When training other model parameters the A-only state parameters are held fixed. Models G1 and G2 are equivalent to Models B1 and B2, although random initial values were chosen prior to training. After training on the sequencing error-corrected data (as described above in Section 4), these models provided further evidence for the lack of ex-tail states (with an approximate 7:3 A:U ratio) in the CO1B, CO3B, and CO3P samples. In Models G3 and G4, state 2 has converged to an ex-tail adding state for the CO1P, ND1B, and ND1P datasets. Interestingly, the unstructured models also suggest a surprising sub-population of tails which immediately enter an ex-tail state without the prior addition of longer U- and A-homopolymers, which we discuss below. An unanticipated discovery such as this reveals the value of unstructured models in this work. For the unstructured models, G5, with 5 emissive states, was the highest complexity that we examined. It has 53 free parameters. They are of high enough dimension that the Baum-Welch training algorithm does not always converge to the global optimum. This increase in local optima results in a difficulty in drawing consistent inferences about the connections to actual biological states in this model. Therefore, we conclude that models with more parameters than Model G5 can have little relevance, at least in this two-observable emission system.

**Fig 14:**
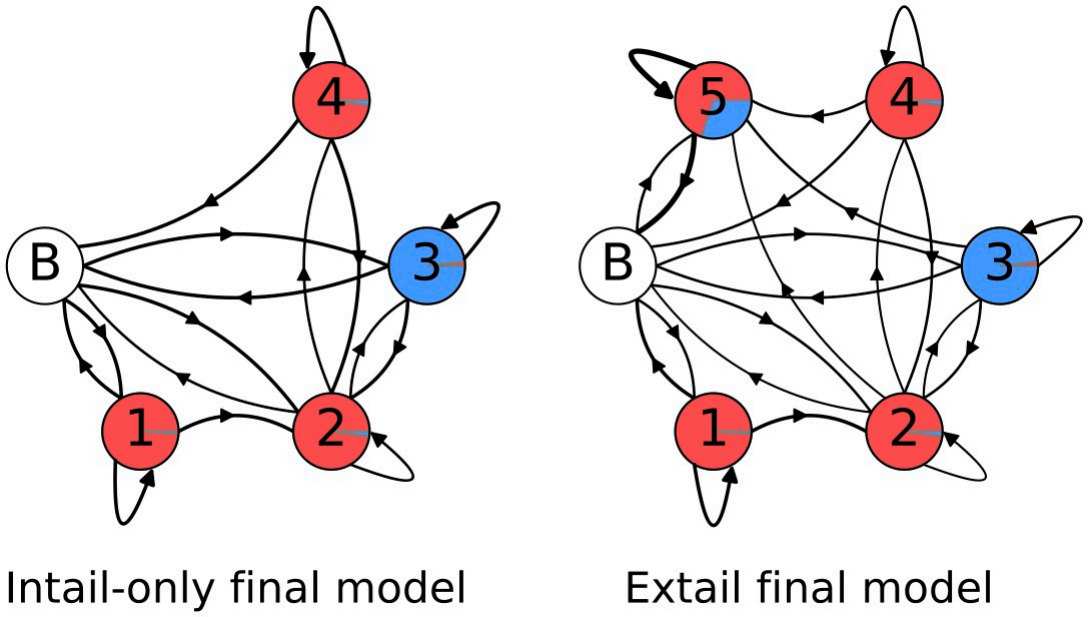
Final model topologies. Pre-training model topologies and emissions for the best unstructured models for each tail dataset. The areas covered by the separate colors in each state circle are proportional to their emissions: uncolored circle labeled ‘B’ indicates the beginning/end state, red is a single adenine addition, and blue is a single uracil addition.

Finally, working off the unstructured models and the emissions that are presented in Fig 13, we added constraints that delineate the A-homopolymer only state suspected earlier to be a discrete entity (Fig 4). As it was clear that even an unstructured model identifies an ex-tail state when it exists, we customized one model for tail populations from transcripts that should be comprised of only in-tails, and one for populations consisting of both in-tails and ex-tails. The basic connectivity and model-to-biological state correspondences of our final consensus models before training (initial values) are shown in Fig 14, a 4-state model for tail populations of in-tails only and a 5-state model for populations containing ex-tails. In both, state 1 was constrained to have a self-transition matching that of a model trained on A-only tails, and state 1 is only accessible by transitions from itself or the initial (B) state. It is unclear *a priori* how exactly to separate the A-only state 1 and state 2 in training the models, and so it is impossible to distinguish their separate functional roles. This ambiguity corresponds to the biological question of how separate in location, composition, and regulation the process of A-only addition is from the A-and-U in-tail addition. Thus states 1 and 2 remain hybrids of the two possible biological pathways, one of which may only add As, and the other which transitions into the A- and U-adding process.

**Fig 15:**
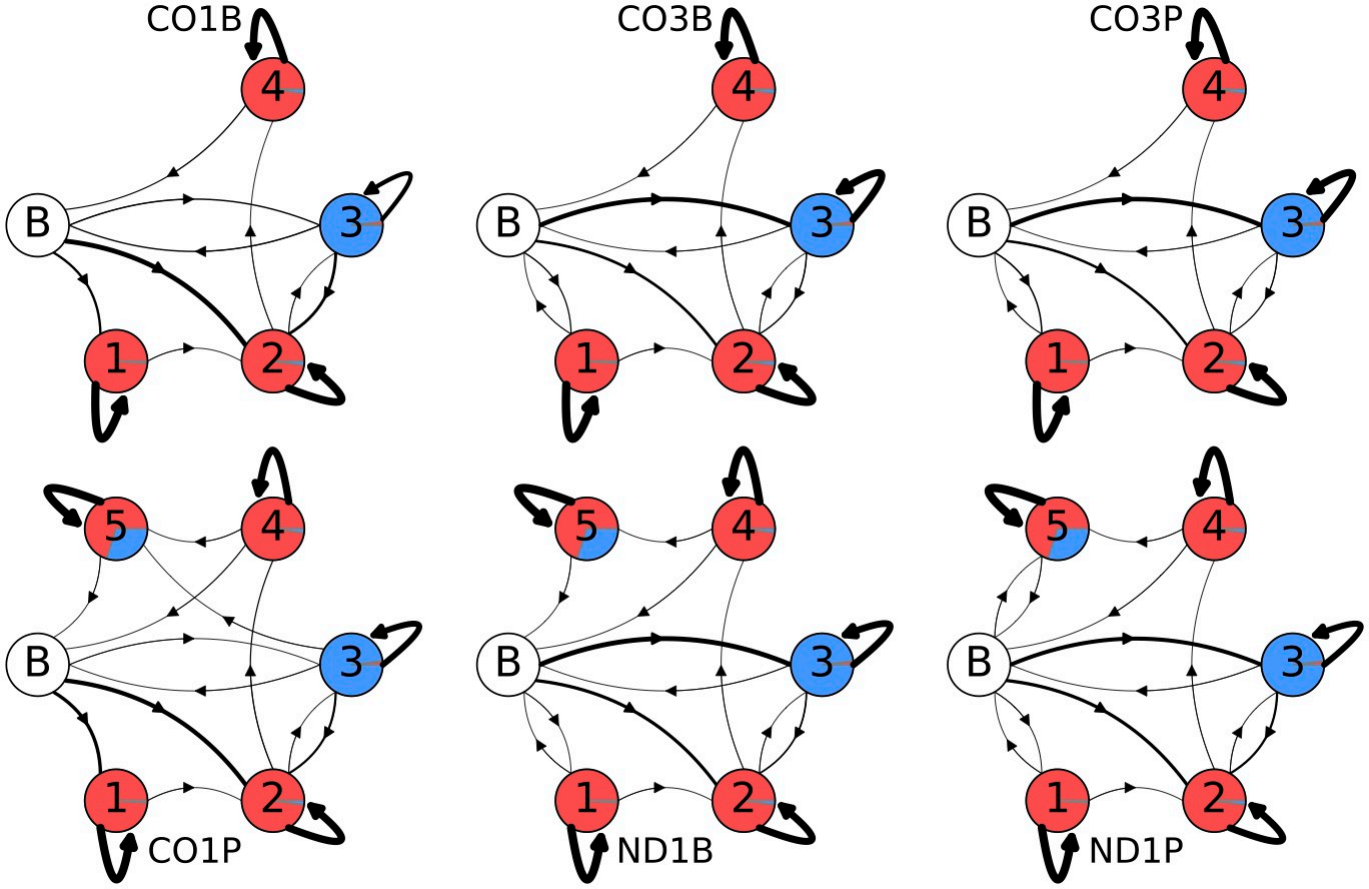
Post-training final models. Post-training model topologies and emissions for the best unstructured models for each tail dataset. The three models in the top row are in-tail only models, while the three bottom row models include ex-tails. The areas covered by the separate colors in each state circle are proportional to their emissions: uncolored circle labeled ‘B’ indicates the beginning/end state, red is a single adenine addition, and blue is a single uracil addition. Line thickness indicates transition probability, with thicker arrows indicating higher probability and thinner arrows indicated lower probability.

After training, the random models with these constraints converge to those shown in Fig 15. The models readily show qualitative differences in emissions of tail populations between the mRNAs and between life-stages. An exception is the tail dataset pair CO3B/CO3P, whose models have trained into very similar forms. As tails are known to play regulatory roles, this indirectly suggests that the pre-edited CO3 mRNA is less differentially regulated between lifestages.

The small numbers of U-additions present in states 1 and 2 in Fig 15 are presumably the result of sequencing and replication errors which introduced spurious Us. While it would be possible to use our trained models to remove these, circular data modification has the potential to distort and overfit it. Therefore, we did not attempt to correct these minor aberrancies.

In general these final models provide an improved fit to the data as determined by the degree to which log-likelihood per emission matches actual values obtained for tail populations. For example, compared to our model B5 the final models improve the log-likelihood per emission by a mean of 1% (this is the total log-likelihood of all sequences from the forward-backward algorithm divided by the total number of nucleotides in the sequences). The most extreme difference in model emissions between states can be clearly observed in Fig 15 between CO1 bloodstream and procyclic form tail populations. Since only the CO1P tail population has tails in an ex-tail state, the CO1 models have differing total numbers of states (4 versus 5). Less visually obvious is that both CO1 samples have the largest proportion of A-only state 1. This is detectable in Fig 15 as the narrower line representing a lower transition probability from the beginning state B to the U-adding state 3 in CO1 models compared to ND1 and CO3. With a lower probability of tails initiating with U, it follows that a higher proportion of tails will feed into the predominantly A-only state 1 (slightly thicker line for CO1).

Additional ways to view the accuracy of final model versus prior model emissions are to compare plots generated with the model outputs with plots of the actual training data. In this and previous work we have plotted directly from the data such features as tail length profiles, homopolymer composition across the tail lengths, overall U and A homopolymer profiles, and the homopolymer profile of the first A homopolymer in tails initiating with “A” (A1). The latter metric is a simple one for which we have already compared the training data set with model B3 emissions in Fig 11 (left two panels). We therefore decided to select this metric to compare the relative abilities of the B2 and final model emissions to predict A1 homopolymer length profiles for the tail populations. The A1 homopolymer length is a parameter capturing both compositional and length data, so it seemed a relevant tool to compare models. A significant improvement is seen for shorter homopolymers. However, the ends of the distributions are not dramatically better, and in general the HMM framework is not optimal for modeling the termination of nucleotide addition. This modeling difficulty suggests that a separate biological mechanism may exist for termination. The same problem exists for the B3 models. When taken in sum, the shape of the A1 length profile curves of the final model (Fig 11 right panel) more closely align in shape and amplitude with those of the training data than those of the B3 model. Some discrepancies, however, still exist.

Finally, ND1 tail population emissions best exhibit the unexpected feature of immediate ex-tail states (state 5), particularly in ND1P tails where the transition probability from the initial ‘B’ state to state 5 is 0.045. We consider this to be an additional advantage of the final models. Tails evidently entirely generated in an ex-tail state may reflect a real difference in regulation. In other words, some ND1 transcripts in the procyclic life stage may bypass the expected in-tail addition stages.

## 6 Conclusion and future application

The 3’ mRNA tail addition system in *T. brucei* mitochondria provides an excellent opportunity to study the application of probabilistic modeling to elucidating genetic and biochemical details of a complex system. Sequence data from this system consists of binary strings which are relatively simple to characterize. Structured HMMs with small numbers (2-4) of hidden states performed well at state classification tasks on these datasets, but failed as generative models. The unstructured state models provided new, testable hypotheses on more subtle variations in tail addition that should correspond to distinct and yet unidentified biological states. For example, we may hypothesize that these variations reflect subtle functional changes to enzymatic or regulatory proteins that result from post-translational modifications, or changes in composition to protein or RNA-protein complexes. Additionally, our added constraints to the unstructured models to reflect the A-only addition pathway more clearly revealed the differences between the datasets in both state transition probabilities and relative nucleotide compositions that define each state post-training. The clarity of modeling output shown here for trypanosome mitochondrial mRNA tail addition demonstrates why it could serve as a real-world introduction to the application of HMM for biological systems in a most simplified form.
